# Smart Consumption, Less Waste: The Role of Digital Technologies in Consumer Food Waste Reduction—A Systematic Literature Review

**DOI:** 10.3390/foods15183228

**Published:** 2026-09-12

**Authors:** Paula Karina Salume, Marcelo Werneck Barbosa, Marcelo de Rezende Pinto

**Affiliations:** 1 Departamento de Ciências Administrativas e Contábeis (DECAC), Universidade Federal de São João del-Rei (UFSJ), Av. Visconde do Rio Preto, S/n, bairro Colônia do Bengo, São João del Rei 36301-360, MG, Brazil; paulasalume@ufsj.edu.br; 2 Department of Agricultural Economics, Pontificia Universidad Católica de Chile, Avenida Vicuña Mackenna 4860, Macul, Santiago 7820436, Chile; 3 Center for Advanced Transportation, Logistics, and Economic Competitiveness (CATLEC), Av. Vicuña Mackenna 4860, Macul, Santiago 7820436, Chile; 4 Programa de Pós-graduação em Administração, Pontifícia Universidade Católica de Minas Gerais (PUC Minas), Av. Dom José Gaspar, 500, Coração Eucarístico, Belo Horizonte 30535-901, MG, Brazil; marcrez@pucminas.br

**Keywords:** food waste prevention, consumer behavior change, technology-driven interventions, sustainable consumption, systematic literature review, technology adoption barriers

## Abstract

Digital technologies are increasingly used to understand and influence consumer food waste behavior, yet evidence on their applications and effectiveness remains fragmented. This systematic literature review synthesizes research at the intersection of consumer behavior, technological innovation, and food waste. Peer-reviewed articles published in English were identified through searches of Scopus and Web of Science, resulting in a final sample of 22 studies. Thematic analyses were conducted to examine publication patterns, research contexts, methodological approaches, and emerging research gaps. The literature shows a shift from predominantly theoretical and exploratory work towards more applied, technology-centered research. Scientific production is concentrated in Australia, China, Italy, the Netherlands, and the United Kingdom, while the selected studies are mainly published in journals focused on environmental sustainability, food-chain management, and consumer psychology. Two broad research approaches were identified: quantitative studies using cross-sectional surveys and statistical modeling, in which technology is treated as an explanatory factor, and experimental or design-oriented studies that employ technology as a direct intervention. However, the evidence base is constrained by extensive reliance on self-reported data, recall and social-desirability bias, and cross-sectional designs that limit causal and long-term conclusions. Future research should therefore prioritize longitudinal, mixed-methods, and objective measurement approaches, while examining emotional and psychological responses to food-waste-prevention technologies. The implementation of such technologies also requires attention to usability, user fatigue, privacy and ethical concerns, and psychological and cultural barriers to adoption. Overall, the findings indicate that digital technologies offer promising but insufficiently validated opportunities to support food waste reduction and that stronger empirical evidence is needed to guide their effective and responsible development.

## 1. Introduction

Food waste (FW) is among the most urgent contemporary challenges, with profound implications for food security, the global economy, and environmental sustainability. Estimates indicate that roughly one-third of all food produced for human consumption is lost or wasted annually, about 1.3 billion tons [1]. This phenomenon not only aggravates food insecurity in a world of finite resources but also substantially contributes to greenhouse gas emissions, inefficient water use, and soil degradation, posing a moral and ethical paradox in the face of global hunger [2].

Several FW reduction initiatives and interventions have been implemented and investigated with different outcomes, with consumer-oriented behavioral interventions generally demonstrating effectiveness [3]. Frequently examined strategies include awareness and information campaigns, tray-less dining, and portion-size reduction [3]; adjustments to plate size, economic incentives, and food sharing on university campuses, although most available evidence originates from developed countries [4]; prompts, self-recording, feedback on previous behavior, educational programs, and practical tips [5]; and tailored campaigns, websites, applications, and educational courses that strengthen young adults’ knowledge, motivation, and food-management skills [6,7]. Such interventions are most effective when practical measures are combined with informational strategies [8], since informational campaigns implemented in isolation may be insufficient unless complemented by environmental or contextual changes that make alternative behaviors easier to maintain [9,10]. This pattern is illustrated across several national contexts: research has particularly focused on the United States, Italy, and the United Kingdom, where consumption accounts for a substantial share of FW and human-development levels are high [11]; examples include the UK’s Love Food Hate Waste campaign, associated with substantial reductions in avoidable household FW and economic savings [12], smaller buffet plates in Norway that reduced plate waste [13], the range of interventions reviewed by Reynolds et al. [14] spanning plate-size modifications, information campaigns, and revised school nutritional guidelines, concise printed prompts that reduced U.S. college students’ FW by 15% [15] and a virtually delivered educational intervention that decreased household FW by almost 28% [16], and a combined education, skills-development, and whole-of-school intervention in Australia that increased student engagement and reduced avoidable school FW [17].

Collectively, these non-digital interventions share a common conceptual limitation: they largely treat the consumer as a rational, cognitively autonomous decision-maker whose behavior is shaped primarily by awareness, motivation, and social norms, an assumption consistent with the Theory of Planned Behavior (TPB). As digital technologies become embedded in food-related routines, they introduce a qualitatively different mechanism of behavior change. Rather than simply informing or motivating consumers, tools such as mobile applications, smart sensors, and AI-based feedback systems actively reconfigure the material and situational context in which food decisions are made.

For the purposes of this review, digital technologies are defined as software- or hardware-based tools that either (a) mediate consumer food-related decisions in situ (e.g., mobile apps, smart bins, IoT sensors), or (b) generate or process data about consumer food waste behavior (e.g., computer vision, machine learning classifiers, mobile applications). This definition is aligned with previous research [18,19,20,21]. Food waste can be understood as the loss of edible products that were originally meant to nourish people but end up being thrown out, spoiled, or otherwise removed from the human food supply before they are actually eaten [22]. This phenomenon spans multiple stages, from harvesting and processing to retail and, ultimately, the moment food reaches consumers [23].

Consumer FW centers on what happens once food has reached the household or end-user level. It captures situations where individuals or families discard items that could still have been eaten, whether because the food spoiled, was over-prepared, went past its use-by date, or was simply left uneaten on a plate [24]. Importantly, this type of waste is shaped heavily by human behavior: the routines, habits, and choices consumers make in managing their food supply play a central role in determining how much ends up being wasted [25]. Some scholars further clarify that consumer-level waste includes not only what is sent to landfill but also food that is composted, fed to animals, or disposed of through household drains; essentially, any pathway that removes edible food from human consumption at this final stage of the supply chain [12]. Genuine consumer FW, then, refers specifically to those instances where something that could have nourished someone is instead lost, discarded, or repurposed for non-food uses by consumers [26]. Household FW is defined as food discarded by consumers at the storage, preparation, or consumption stages within the home, as opposed to waste occurring in retail, food service, or supply-chain stages [1,27].

Emerging technologies offer additional possibilities for food sharing, inventory management, recipe recommendations, and the reuse of leftovers through smart systems, online platforms, and mobile applications [9,28]. Consumers tend to favor simple, accessible, and technology-supported campaigns, including applications offering recipes and reward-based features; nevertheless, relying on information about environmental benefits alone may not be sufficient to change behavior [29]. Overall, technology can support FW reduction by facilitating food planning, inventory management, leftover reuse, food sharing, and the delivery of tailored information. However, previous interventions have generally treated technology as a complementary communication tool rather than a central component of the reduction strategy. Consequently, its potential to combine practical support, behavioral feedback, and context-sensitive solutions remains insufficiently explored.

Within this context, FW consumer behavior emerges as a critical intervention point. In both developed and developing countries, a substantial share of waste occurs at the final stages of the supply chain, with households accounting for more than half of total waste in some regions such as the European Union [30,31]. The drivers of this behavior are multifaceted, ranging from limited planning and cooking skills to confusion over date labels and a preference for visually perfect products, requiring analysis that moves beyond quantitative metrics to address the psychosocial and routine dimensions of consumption [32]. Also, FW is a topic of interest to consumers that has been discussed in social media, including initiatives that foster FW reduction and promote leftovers management [33].

Research on FW has traditionally drawn on economic, psychological, and sociological perspectives, frequently employing the TPB to explain individuals’ intentions and actions [31,34]. TPB remains the most commonly applied theoretical framework for understanding consumer determinants in this field [24,32,35,36,37]. However, purely cognitive approaches have proven insufficient to capture the complexity of everyday practices that lead to food disposal, indicating the need to incorporate situational and material factors into analyses [31]. Recent literature highlights the importance of examining how the domestic environment and available tools shape discard decisions, shifting attention from consumer blame toward systemic barriers and opportunities for change [18].

Several literature reviews have sought to systematize knowledge on the determinants of FW [31,32,36,37,38,39]. Hebrok and Boks [31] conducted an extensive review focused on drivers and design intervention points, emphasizing the complexity of consumption practices and the need for solutions that go beyond information provision. Moraes et al. [2] categorized prevention and minimization methods, such as education, policy, and logistics, while noting the scarcity of empirical studies assessing the effectiveness of these measures. More recently, Vihttuari et al. [32] reviewed the literature, identifying levers for behavioral change. Despite these contributions, a persistent gap remains: technology is often treated as peripheral or subsumed under opportunity or infrastructure, rather than examined as a central, transformative agent in contemporary consumption practices [2,32]. A bibliometric analysis by Salume et al. [36] corroborates this view, acknowledging the field’s maturation while calling for more integrated approaches and methodological innovation. Most prior reviews have not foregrounded technology as a primary analytic focus, thereby overlooking how digital tools and material innovations are reconfiguring consumers’ relationships with food.

The growing role of technology in this domain is ambivalent and transformative. On one hand, food delivery applications have been associated with increased waste through stimulated over-purchasing and difficulties in managing leftovers [35,40]. On the other hand, innovations such as smart packaging, freshness sensors, and household inventory management apps promise to extend product shelf life and support consumer decision-making [41,42]. Additionally, novel research methodologies that leverage technology, such as technology probes and artificial intelligence (AI) for image-based waste analysis, are emerging as more accurate means to quantify waste and uncover underlying behaviors, thereby overcoming limitations of self-report measures [18,43,44]. Thus, technology functions both as an intervention tool and as a vector that fundamentally alters consumption practices and the scholarly study of FW. Also, there has been research on FW reduction strategies supported by digital technologies in the retail, services, and hospitality sectors [20,21]. However, research focusing on the use of digital technologies and consumer FW remains scant.

Given this landscape, the present study aims to understand the role of technologies in research on consumer FW. The objective is to map how technological solutions are employed both to mitigate waste and to deepen academic understanding of wasteful habits. The central research question guiding this investigation is “How are technological solutions being used in studies of consumer food waste?” To address this question, a Systematic Literature Review (SLR) was conducted to identify, evaluate, and interpret all relevant research, following the guidelines of Tranfield et al. [45] and adaptations used in recent studies in the field [36]. The SLR was structured around three research questions (RQs):

- RQ1: What is the overall landscape of publications on consumer FW and technology, considering temporal trends, geographic distribution, and leading journals?
- RQ2: Which methods, behavioral theories, and technologies are predominantly used to study consumer FW, and how have they transformed research on the topic?
- RQ3: What are the main gaps in the current literature and what future research agenda can advance knowledge at the intersection of consumer FW and technology?

This study aspires to deliver contributions across multiple dimensions. Theoretically, it seeks to advance understanding of the intersections among technology, consumer behavior, and FW by proposing a more holistic perspective that incorporates technological materiality and consumption behavior. This study systematically integrates three domains that have often been treated separately—consumer behavior, technological innovation, and food sustainability—by offering a conceptual and methodological map of the state of the art. By making explicit the dominant theoretical approaches, the technologies employed, and the persistent gaps, the review guides researchers in identifying promising avenues for inquiry, designing more rigorous studies (notably those incorporating objective measurements), and formulating future research agendas that are more integrated, comparative, and longitudinally grounded [39].

Methodologically, the paper highlights opportunities to refine or propose methods for studying this intersection, showing how tools such as AI and participatory visual methods can help overcome social-desirability and other biases common in food waste research [43,44]. Practically, the review aims to inform the development of more effective technologies and interventions by identifying which design features and functionalities in apps and smart devices are most likely to foster engagement and behavioral change [3]. Finally, from a public-policy perspective, the findings can support the design of strategies to reduce FW, indicating how governments might incentivize the adoption of circular-economy technologies as services and regulate digital platforms to mitigate their negative impacts [40,42].

## 2. Materials and Methods

This research was conducted as an SLR, a method that, unlike traditional narrative reviews, emphasizes rigor, transparency, and replicability to synthesize scientific evidence while minimizing bias. The SLR approach is particularly suited to manage fragmented knowledge and to identify critical gaps in multidisciplinary fields that intersect technology and sustainability [45,46]. The review followed the PRISMA (Preferred Reporting Items for Systematic Reviews and Meta-Analyses) guidelines to ensure methodological rigor, transparency and reproducibility. The PRISMA framework [47,48] provided a 27-item checklist and a flowchart to document the stages of identification, screening, eligibility and inclusion, thereby standardizing the review process and reducing reporting bias.

The methodological workflow comprised four main phases: Planning, Identification of evidence, Screening and eligibility, and Data synthesis, following the recommendations of Tranfield, Denyer and Smart [45] and Snyder [49], and incorporating the procedural flow of PRISMA 2020 [47]. During planning, the review scope and research questions (RQ1–RQ3) were defined to focus the search strategy on the intersection between consumer food waste and technological solutions.

The following inclusion criteria were defined: (a) peer-reviewed journal articles; (b) texts published in English; and (c) studies explicitly addressing the application of digital technologies in the context of consumer FW. The concept of digital technologies in this study includes, for instance, studies in which technology is used in an actual intervention, a consumer-facing platform, or an AI-assisted analytical tool. Exclusion criteria eliminated book chapters, conference abstracts, documents without full text, duplicates and publications in languages other than English. Gray literature and conference summaries were excluded to prioritize peer-reviewed evidence and ensure the robustness of synthesized findings. Book chapters and conference abstracts were excluded because they often lack the peer review, methodological, and outcome detail required to assess study quality, risk of bias, and reproducibility. Conference abstracts may report preliminary findings that differ from subsequent full publications [50,51], may not provide sufficient information to identify study designs or extract outcomes for synthesis, and can be difficult and resource-intensive to locate and evaluate because of inconsistent indexing [52,53,54]. Although discrepancies between abstracts and full articles are generally minor, abstracts commonly present preliminary results [55], whereas book chapters may contain narrative or previously published material rather than original empirical research. These sources were therefore excluded a priori to ensure consistency in the appraisal and synthesis of fully reported primary studies, while acknowledging the potential for publication bias.

A systematic search was performed in the electronic databases Scopus and Web of Science (WoS), which were selected for their comprehensive coverage of research on consumer behavior, technology, and sustainability. Also, they were selected because they are widely recognized, comprehensive bibliographic databases that primarily index scholarly journals and provide relatively accurate, structured, and reproducible records [56,57,58]. Their extensive but partially complementary coverage across scientific domains, particularly the broader representation of social sciences in Scopus and of natural sciences and engineering in WoS, allows their combined use to provide a richer range of relevant literature [59,60]. Both databases are also regarded as reliable sources for handling large volumes of academic information and for supporting systematic searches and research analyses [61,62,63]. Although their coverage is subject to country, language, and disciplinary biases, they were considered appropriate for identifying studies published in established, indexed scholarly journals [59].

The search combined exhaustive terms related to consumer behavior, FW, technologies (e.g., apps, IoT, sensors, AI, machine learning, computer vision, smart packaging), and relevant settings (e.g., household, restaurant, institutional food service). Boolean operators and wildcards were applied, and no temporal limits were imposed to capture historical evolution and emerging trends.

The search strategy used the following combination of terms, which were adapted to the specific syntax of each database. In WoS, the following string was used—TS = (“consumer behavio*” OR “consumer attitude*” OR “consumer decision*” OR “consumer choice*” OR “consumer awareness”) AND TS = (“food waste” OR “food loss” OR “food discard*” OR “food surplus” OR “food wastage” OR “wasted food”) AND TS = (technolog* OR digital* OR app* OR “mobile app*” OR smartphone* OR “Internet of Things” OR IoT OR sensor* OR “smart kitchen” OR “smart appliance*” OR “smart fridge*” OR “computer vision” OR “image recognition” OR “machine learning” OR “artificial intelligence” OR “deep learning” OR “recommender system*” OR “recommendation system*” OR “smart label*” OR “time-temperature indicator*” OR “time temperature indicator*” OR TTI OR “freshness indicator*” OR RFID OR “QR code*” OR barcode* OR “digital nudg*” OR gamif* OR chatbot* OR “virtual assistant*”) AND TS = (household* OR home* OR resident* OR “end user*” OR “lay user*” OR restaurant* OR “food service” OR canteen* OR cafeteria* OR school* OR hospital* OR “university dining” OR “institution*” OR “plate waste”).

In Scopus, the following string was used—TITLE-ABS-KEY (“consumer behavio*” OR “consumer attitude*” OR “consumer decision*” OR “consumer choice*” OR “consumer awareness”) AND TITLE-ABS-KEY (“food waste” OR “food loss” OR “food discard*” OR “food surplus” OR “food wastage” OR “wasted food”) AND TITLE-ABS-KEY (technolog* OR digital* OR app* OR “mobile app*” OR smartphone* OR “Internet of Things” OR IoT OR sensor* OR “smart kitchen” OR “smart appliance*” OR “smart fridge*” OR “computer vision” OR “image recognition” OR “machine learning” OR “artificial intelligence” OR “deep learning” OR “recommender system*” OR “recommendation system*” OR “smart label*” OR “time-temperature indicator*” OR “time temperature indicator*” OR TTI OR “freshness indicator*” OR RFID OR “QR code*” OR barcode* OR “digital nudg*” OR gamif* OR chatbot* OR “virtual assistant*”) AND TITLE-ABS-KEY (household* OR home* OR resident* OR “end user*” OR “lay user*” OR restaurant* OR “food service” OR canteen* OR cafeteria* OR school* OR hospital* OR “university dining” OR “institution*” OR “plate waste”).

In these strings, it is necessary to explain that (1) TS (Topic) or Title-Abs-Key searches for terms in the title, abstract, and keyword fields; (2) OR and AND are Boolean operators that combine search terms; (3) * is a wildcard character that allows the inclusion of variations of a word (for example: “behavior” and “behaviors”).

The initial search returned 489 records (195 from WoS; 294 from Scopus). Technical filters removed 81 non-article documents (23 WoS; 58 Scopus) and 5 non-English studies from Scopus, yielding 403 articles for further processing. After de-duplication (151 records removed), 252 unique records remained for title and abstract screening.

Three researchers independently screened titles and abstracts to verify the presence of technological components. Inter-rater agreement was high, and any disagreements were resolved through discussion until consensus. This stage excluded 218 records, leaving 34 articles for full-text review. Kappa values or other quantitative measures for inter-rater agreement were not calculated at this stage because of the high number of excluded records and the risk of kappa paradox [64], which may reveal a low kappa value even when very high agreement in percentage terms is seen [65].

Full-text eligibility assessment of the 34 retained articles was conducted exclusively by the three authors, each of whom independently evaluated each article against the inclusion/exclusion criteria. Inter-rater agreement was assessed using Fleiss’ kappa across the reviewers (κ = 0.87), indicating substantial agreement; remaining disagreements were resolved through discussion until consensus, resulting in a final sample of 22 studies.

Following completion of the screening process, NotebookLM Pro (now Gemini Notebook) was used solely as a synthesis-support tool to help organize and cross-reference descriptive information extracted from the 22 included studies (e.g., summarizing reported methods, theories, and technologies). All AI-generated summaries were manually checked by the authors against the original full texts for inaccuracies and corrected before being incorporated into the analysis. Any discrepancies identified between AI-generated summaries and the source text were resolved by consensus among the three authors, with the original full text always treated as the authoritative source. The AI tool was not used to inform or make inclusion/exclusion decisions.

**Figure 1:**
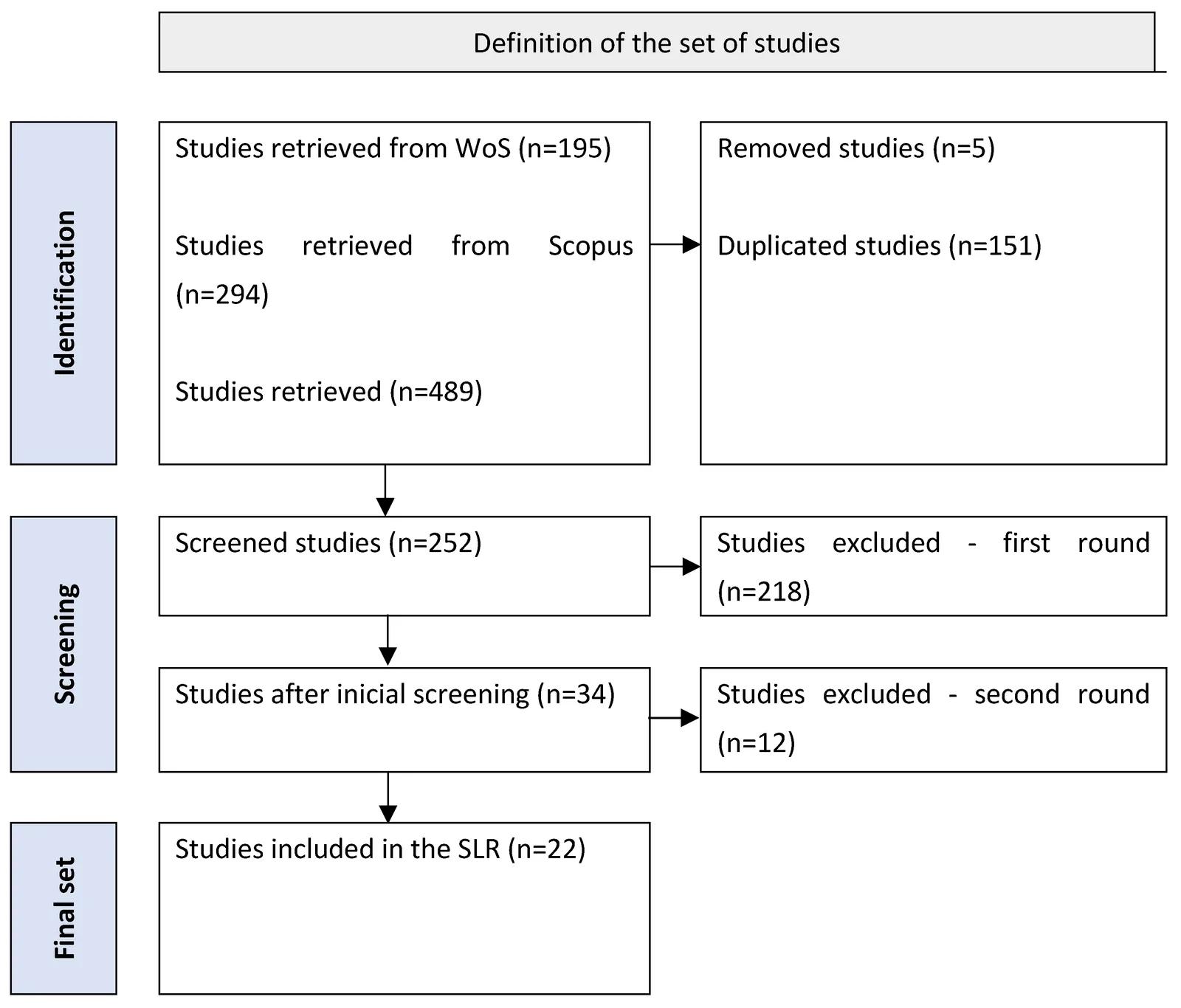
PRISMA flowchart of this systematic literature review. Source: Prepared by the authors, adapted from Page et al. [47].

The PRISMA 2020 [47] flow diagram, summarizing the identification, screening, eligibility, and inclusion steps, is presented in Figure 1.

The final sample of 22 studies reflects the strict application of the inclusion/exclusion criteria rather than an arbitrary target. This is consistent with the nascent stage of research explicitly combining digital technologies with consumer food waste behavior, and comparable in size to other focused SLRs in adjacent fields (e.g., [32,66,67,68,69]). Given that SLRs routinely exclude the majority of initially identified records due to strict eligibility criteria [70,71], a compact but rigorously screened sample is appropriate for synthesizing this emerging literature.

Data extraction from the selected studies followed a mixed approach. Descriptive bibliometric mapping was performed using the Bibliometrix package via the Biblioshiny interface (RStudio—version 4.5.1), enabling structured visualization of bibliographic metadata in line with recent reviews in the FW field [32,36]. The integrative synthesis consolidated evidence through a staged procedure. First, a descriptive mapping documented temporal publication trends, the geographic distribution of research hubs, and leading journals, providing contextual orientation. Next, thematic analysis categorized research methods, behavioral theories and predominant technologies, revealing how digital and intelligent tools have shifted traditional approaches toward real-time interventions and more precise data collection. Finally, a critical appraisal identified persistent theoretical and methodological gaps. These omissions directly informed the proposed future research agenda, thereby transforming raw data into a consolidated, forward-looking perspective that fulfills the study’s objectives.

To characterize the methodological profile of the 22 included studies without altering the final sample, a quality appraisal was conducted using the Mixed Methods Appraisal Tool (MMAT) [72]. MMAT was selected because it accommodates the methodological diversity of the included literature, spanning qualitative, quantitative descriptive, quantitative non-randomized, randomized controlled trial, and mixed-methods designs, within a single, validated instrument. Each study was first classified into one of MMAT’s five study-design categories and screened against two universal criteria (clarity of research questions and adequacy of data to address them), followed by five category-specific criteria rated as “Yes,” “No,” or “Can’t tell” based on the full text of each article.

Because MMAT’s five design-specific criteria sets presuppose primary data collection from human participants, they could not be applied to the two computational or algorithmic studies [73,74]. However, the two universal MMAT screening questions (S1–S2), which are design-agnostic, were retained for these studies, and the five design-specific criteria were substituted with four adapted criteria (C1–C4) tailored to computational research (see Appendix B). The four adapted criteria (C1–C4) applied to the two computational/optimization studies [73,74] are grounded in Sargent’s verification-and-validation framework for simulation models [75], which distinguishes conceptual model validity, data validity, and operational validity as core dimensions for appraising simulation-based research. This framework was selected because both studies employ optimization/simulation approaches. C1 corresponds to Sargent’s conceptual model validity; C2 corresponds to data validity; C3 corresponds to operational validity; and C4 addresses the boundary conditions for generalizing simulated results to real-world consumer behavior, a dimension Sargent identifies as essential to responsible interpretation of simulation outputs.

Both Ou et al. [74] and Woolley et al. [73] received positive ratings for C1 and C2, reflecting their transparent methodological assumptions and clearly documented data provenance. Both were rated negative for C3, as their reported benefits stem from simulation-based analyses rather than real-world empirical validation: Ou et al. [74] applied a one-year retrospective simulation to historical sales data, while Woolley et al. [73] explicitly note that their tool has not yet been implemented in a real scenario. For C4, Woolley et al. [73] were rated positively due to an explicit discussion of technological, legislative, commercial, and social barriers to implementation, whereas Ou et al. [74] received a CT rating owing to the absence of a dedicated limitations discussion.

Consistent with MMAT developer guidance, ratings were not aggregated into a composite numerical score, nor were they used as a basis for excluding studies from the review. Rather, the appraisal was conducted after the final sample of 22 studies had been established through the inclusion/exclusion criteria described above and served exclusively to systematically document methodological strengths and limitations informing the narrative synthesis presented in Section 3.3 and Section 4.2. Ratings were independently assessed by three reviewers; disagreements, where applicable, were resolved through discussion. The complete appraisal matrices are presented in Appendix A and Appendix B.

All studies met the first universal screening criterion of MMAT (clear research questions). Data adequacy (S2) could not be confirmed with certainty for two studies [73,76]. Among the twelve studies classified as quantitative descriptive designs [35,40,41,42,43,77,78,79,80,81,82,83], the most frequent source of uncertainty concerned sample representativeness and the risk of non-response bias, echoing the sample-limitation concerns discussed narratively above (e.g., homogeneous, convenience-based, or geographically concentrated samples). The single randomized controlled trial [84] satisfied randomization appropriateness and baseline comparability, with confirmed blinding of outcome assessors (C4), although complete outcome data (C3) and full participant adherence (C5) were not met, consistent with documented data exclusions and item-recording deviations in the original study.

The single quantitative non-randomized study [85] met four of the five category-specific criteria, except for participant representativeness relative to the target population (C1 = No), while all other criteria were rated favorably. The single qualitative study [76] satisfied the criterion of methodological appropriateness (C1 = Yes) but showed insufficient substantiation of interpretations relative to the data (C4 = No), with data adequacy, coherence between collection and analysis, and derivation of findings rated as “Cannot tell” (S2, C2, C3, C5). The five mixed-methods studies [18,19,34,44,86] demonstrated adequate rationale and integration of qualitative and quantitative components, though Kör et al. [19] showed inadequate rationale, integration, and interpretation (C1 = No, C2 = No, C3 = No), with divergence-handling unclear (C4 = Can’t tell); only Jones-Garcia et al. [18], Lim et al. [86] and Soma et al. [34] explicitly addressed such divergences (C4 = Yes), while Gul et al. [44] left this unclear (C4 = Can’t tell). The two computational/simulation studies [73,74], appraised using adapted criteria given their non-empirical design, transparently reported model assumptions but rarely included empirical validation against real-world data.

## 3. Results

### 3.1. Overview of Publications

This subsection reports the findings that allow us to address RQ1, which examines the publication landscape on consumer behavior, FW, and technologies in the studies included in the review, focusing on temporal evolution, geographic distribution, and leading journals.

**Figure 2:**
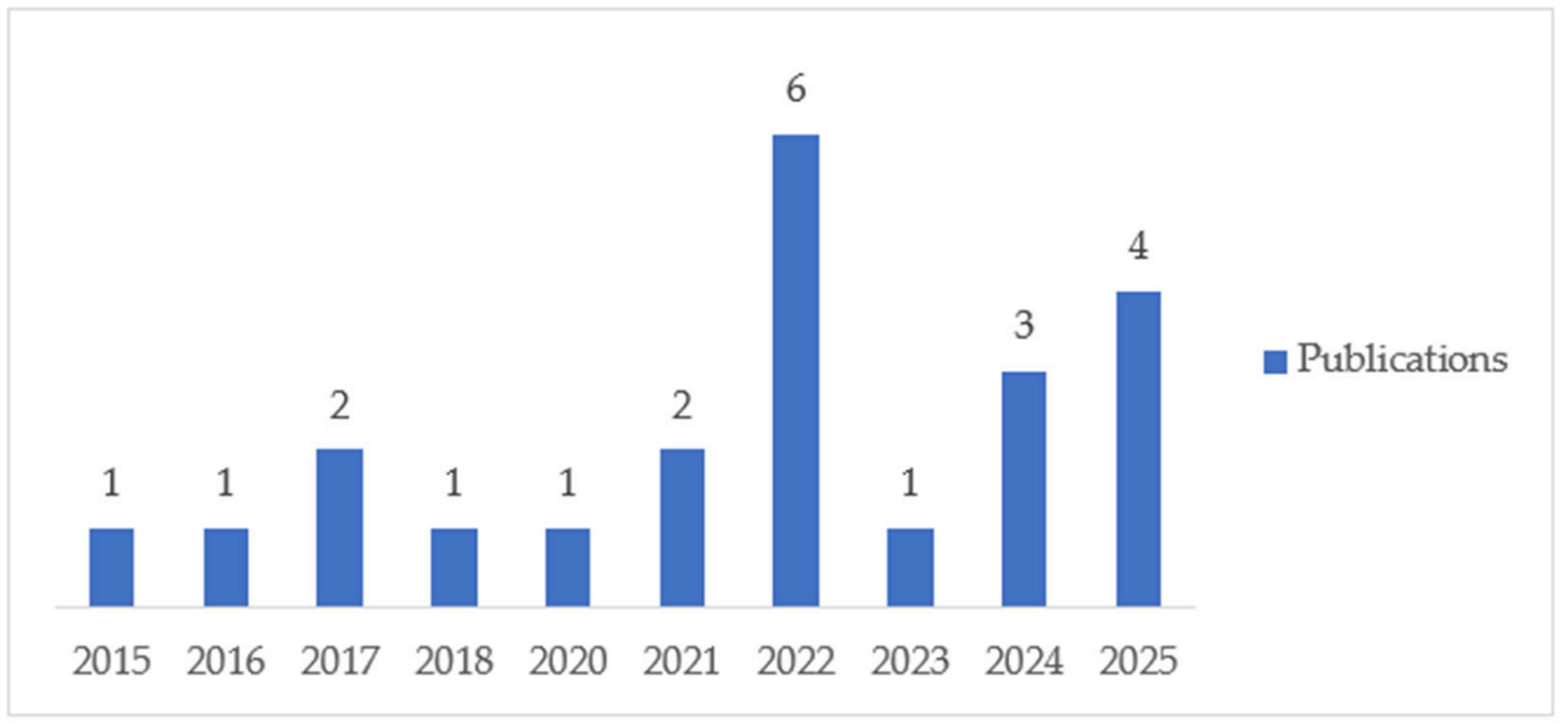
Evolution of publications. Source: Prepared by the authors, based on data from WoS and Scopus.

Figure 2 displays the annual publication counts. Between 2015 and 2018, five studies were published. These early works primarily emphasized theoretical acceptance and observational accounts of behavior. That period marks a shift from intention-based studies toward in situ observation: Jörissen, Priefer and Bräutigam [77] investigated acceptance of smart refrigerators prior to their widespread adoption, while Masson et al. [81] and Lim et al. [86] advanced research into controlled environments and prototype trials (technology probes), acknowledging limitations of self-reported data. Technology at that stage was often framed as a future promise or an educational tool [76].

The transition phase (2020–2022) saw a marked increase, with nine publications. These studies integrated digital technologies more centrally into research designs and revealed three main developments: (1) use of mobile apps for real-time data collection; (2) deployment of collaborative platforms for surplus redistribution; and (3) initial applications of machine-learning algorithms to detect consumption patterns. Sharma et al. [80] and Shankar et al. [35] treated digital convenience as a driver of waste, whereas Roe et al. [84] used apps for personalized coaching, shifting from passive observation to active behavior change. Notably, 2022 accounted for six publications, reflecting consolidation of the techno-behavioral paradigm and coinciding with wider adoption of anti-waste apps and UN food-sustainability guidance.

The current phase (2023–2025) is characterized by technological sophistication and methodological diversification. Eight articles from this triad reveal three emerging trends: adoption of advanced technologies such as blockchain and computer vision; integration of multiple technologies into complex systems (e.g., IoT combined with generative AI); and broader geographic scope with transnational comparative studies leveraging big data. The field has progressed from descriptive models toward predictive and prescriptive approaches: Gul et al. [44] and Yu et al. [43] exemplify studies that both examine technology and employ AI (computer vision, natural language processing) to validate data and overcome self-report bias. Overall, 16 publications were concentrated in the last five years, indicating rapid diversification and increasing technological sophistication in this research area. However, as discussed in Section 3.3, this diversification has not been uniformly accompanied by gains in methodological rigor, since many recent studies continue to rely on small, non-representative samples and cross-sectional or short-duration designs.

The temporal pattern demonstrates a clear trajectory from theoretical and exploratory studies to applied, technology-centered research with increasing capacity for practical intervention. The data suggest that future research will likely emphasize integrated intelligent systems that combine sophisticated behavioral analysis with emergent technologies to deliver scalable solutions for food waste reduction.

The analysis of the selected set of papers identifies five leading nations in scientific output on consumer behavior, FW, and technologies, considering authors’ affiliations: Australia, China, Italy, the Netherlands, and the United Kingdom. A second tier of contributors includes Canada, Japan and Norway.

Table 1 displays the distribution of the selected articles across 15 different journals, underscoring the multidisciplinary and dispersed nature of research at the intersection of consumer FW and technology.

**Figure 3:**
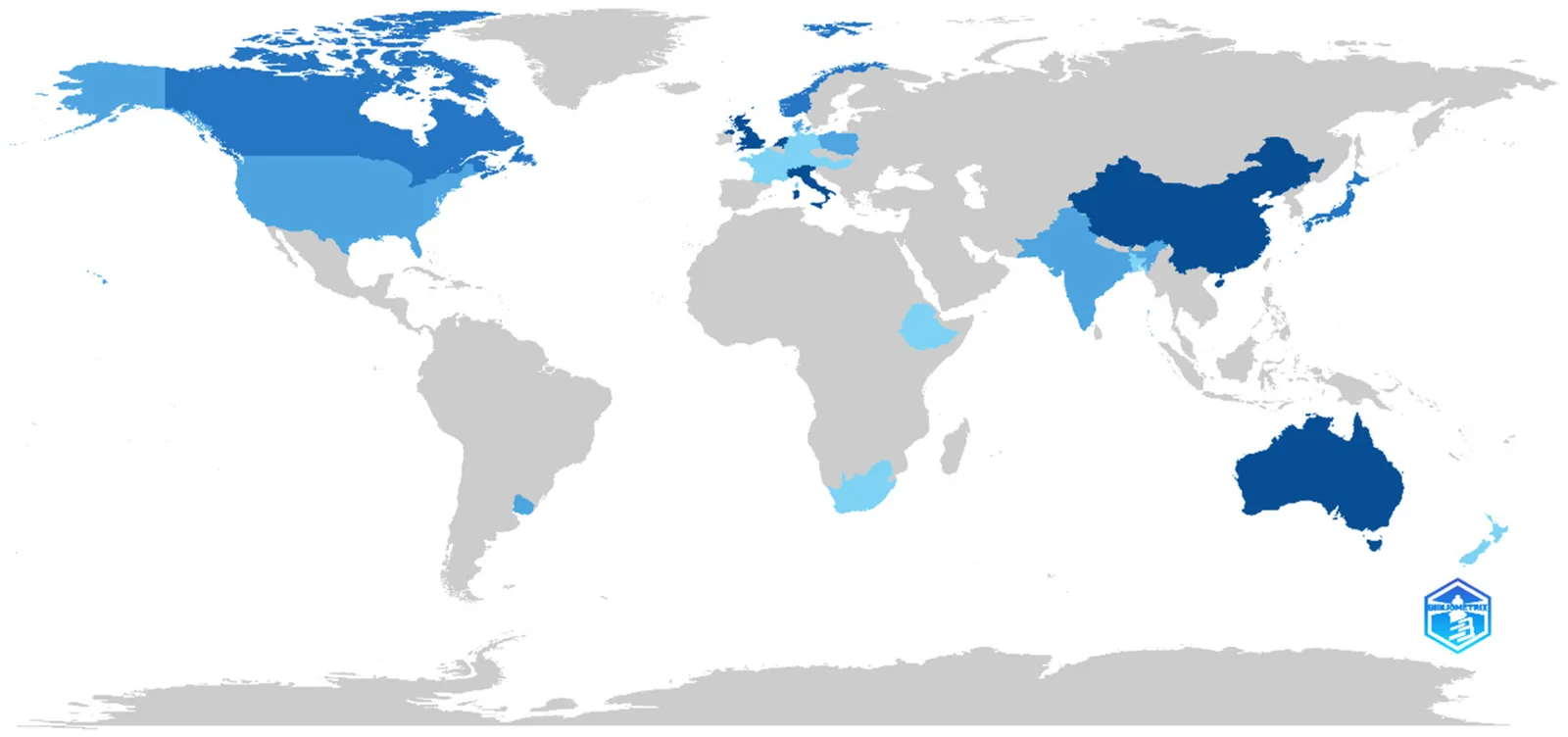
Map of scientific production. Source: Bibliometrix, based on data from WoS and Scopus.

Figure 3 maps global scientific production and international collaboration networks in the field of consumer behavior, food waste, and digital technologies. In this choropleth visualization, the color gradient applied to the countries (shades of red) represents the absolute volume of scientific production, with darker red tones indicating higher publication counts.

Although the articles are dispersed across 15 journals, there is a notable concentration in three outlets that together account for nearly half of the sample (10 articles). *Sustainability* leads with four publications, followed by *British Food Journal* and *Food Quality and Preference*, with three articles each. This clustering indicates that scholarly debate is principally anchored in three interrelated pillars: environmental sustainability, food-chain management, and consumer psychology. The long tail of the distribution, 12 journals with a single article each, demonstrates the topic’s penetration into highly specialized technical and managerial fields.

Although the search strategy deliberately included terms spanning household, hospitality, and institutional food-service settings, the final sample was predominantly composed of studies situated in household/domestic contexts, with a smaller subset addressing institutional or quasi-institutional settings (e.g., university dining in Roe et al. [84]; university-based food redistribution in Wiśniewska and Czernyszewicz [83]). Because household and institutional FW generation involve distinct decision-making structures, actors, and infrastructures, findings across these settings are synthesized thematically with respect to methods, theories, and technology types, but are not treated as directly comparable regarding the magnitude or generalizability of waste-reduction outcomes.

### 3.2. Methods, Behavioral Theories and Technologies Applied to Consumer Food Waste Research

The systematic analysis of the selected studies addressing RQ2 reveals a clear methodological bifurcation in how technology is investigated within consumer FW research. On one side are quantitative investigations, predominantly cross-sectional surveys coupled with statistical modeling, that treat technology as an antecedent variable within theoretical models. On the other side are experimental and design-oriented studies that deploy technology as a direct intervention. The quantitative strand frequently employs structural equation modeling or regression analyses to test the generalizability of behavioral constructs across large samples, as exemplified in studies examining the effects of delivery apps and digital platforms [35,40,80,83]. These approaches are favored for assessing how digital technologies shape attitudes and waste-related behaviors in specific cultural contexts.

With respect to behavioral theory, Ajzen’s TPB remains the dominant framework, often extended to incorporate technological and contextual variables. For instance, Shankar et al. [35] augment the classical TPB constructs (attitude, subjective norms, perceived behavioral control) with measures such as trust in technology and routines for managing leftovers to explain intentions and over-purchasing behavior. Complementing TPB, the Motivation–Opportunity–Ability (MOA) framework has gained traction as a robust lens for capturing behaviors constrained by situational factors; studies by Aloysius et al. [82] and Soma, Li and Maclaren [34] illustrate how technology primarily operates within the “Opportunity” dimension, either facilitating or impeding household waste management.

Beyond these mainstream models, several studies adopt alternative theoretical lenses to capture nuances of human–technology interaction and consumption decision processes. Sharma et al. [80] apply the Theory of Reasoned Action to map pro- and contra-reasons for app use during the pandemic, offering a more granular account of consumer cognitive processing than TPB alone. Islam et al. [40] employ the Stimulus-Organism-Response-Consequence (S-O-R-C) model to trace causal pathways from technological stimulus (app convenience) to environmental consequence (waste), framing technology as an external trigger that alters internal consumer states. Wiśniewska and Czernyszewicz [83] use the FAB (Food Sharing Attitudes and Behaviors) model to decompose sharing propensity into cognitive, affective, and behavioral components.

A distinct research strand departs from variance-based models and embraces practice-theoretical and qualitative, probe-based methods to reveal how technology becomes entangled in everyday routines. Jones-Garcia et al. [18] developed a smart bin equipped with cameras and scales linked to a Raspberry Pi, automatically recording weight and images at each lid opening; this approach both documented routine invisibilities and functioned as a reflexive intervention. Lim et al. [86] similarly used an augmented bin with weight sensors connected to a social-recipe app, demonstrating that objective eco-feedback increased awareness and engagement more effectively than recipe suggestions alone. These interventionist sensor-based methodologies aim not to predict intentions but to observe how technologies render previously invisible waste behaviors visible, thereby exposing discrepancies between self-reported and actual practices.

Finally, the reviewed studies classify the contextual role of technology into three principal categories, the first being technology conceptualized as part of a theoretical model. In such cases, digital apps, smart infrastructure, and intelligent packaging are treated as environmental attributes or hypothetical product scenarios rather than operational tools. For example, Tadesse et al. [41] and Jörissen et al. [77] assess acceptance and willingness to pay for innovations like QR codes and active packaging, presenting technology as a product attribute to elicit preferences and future intentions [19,78]. In related work, Wiśniewska and Czernyszewicz [83] integrate technology into theoretical models as a contextual facilitator, examining platforms and mobile apps (e.g., Too Good To Go) that connect donors and recipients and offering practical recommendations for IT solutions to support food redistribution in university settings.

A second category frames technology as an active intervention, wherein digital tools are deployed to modify behavior in real time. Randomized controlled trials exemplify this approach: Roe et al. [84] implemented the FoodImage app for tracking and coaching, asking participants to photograph their plate waste rather than self-report it; trained researchers coded the images, producing an objective measure that evidenced a 78.8% reduction in plate waste in the intervention group. Yu et al. [43] evaluated the effectiveness of a food-sharing platform (Regusto) by applying AI models to user-submitted photos of restaurant leftovers; algorithmic analysis revealed a critical discrepancy, with consumer visual estimates of waste differing by approximately 50% from AI-derived weight estimates, underscoring the limitations of human estimation and the superior precision of digital quantification. Soma, Li and Maclaren [34] further illustrate how technology can expose intervention shortcomings: their gamified online education increased self-reported awareness but, when validated through physical curbside waste audits, produced no statistically significant reduction in measured waste, highlighting the necessity of independent, objective outcome measures.

The third category treats technology as a methodological instrument for analysis. Advances in computational capacity have enabled the use of AI and machine-learning algorithms to process complex consumer-behavior data. Random Forest models have been used to predict consumption patterns and prioritize circular-economy measures [42,79], while Gul et al. [44] combined computer vision and natural language processing to scale qualitative visual analyses, producing rapid, less biased insights into waste perceptions. Participatory methods were augmented with AI; photovoice images collected from students were automatically classified by neural networks with 88% accuracy, enabling large-scale processing of qualitative data and provoking strong visual feedback among participants [44].

Computational simulation and algorithmic optimization form an additional subcategory: Woolley et al. [73] and Ou et al. [74] developed algorithms to simulate ideal inventory and sales scenarios, proposing technological architectures that, if implemented, would reshape consumer decision structures to optimize consumption and reduce waste. Passive observational technologies have also been employed to overcome social-desirability bias in self-reports. Biometric measures, such as eye-tracking, revealed rapid, low-effort cognitive rejection of visually imperfect produce [85], while detailed behavioral observation using specialized software (e.g., Noldus) allowed Masson et al. [81] to quantify storage errors and exposure times that compromise food safety—data unlikely to be captured accurately through retrospective questionnaires.

Collectively, the reviewed literature documents a methodological shift from intention-based models (TPB, MOA), which are measured via surveys, toward data-driven, interventionist approaches that employ digital interventions (apps, IoT) and advanced analytics (AI, ML). While behavioral theories remain essential to explain adoption and resistance, the integration of digital interventions and analytical tools enables researchers to measure how and to what extent technology affects waste outcomes. Thus, technology has evolved from a theoretical object of study into both an active agent of behavioral change and a sophisticated instrument for scientific inquiry.

**Figure 4:**
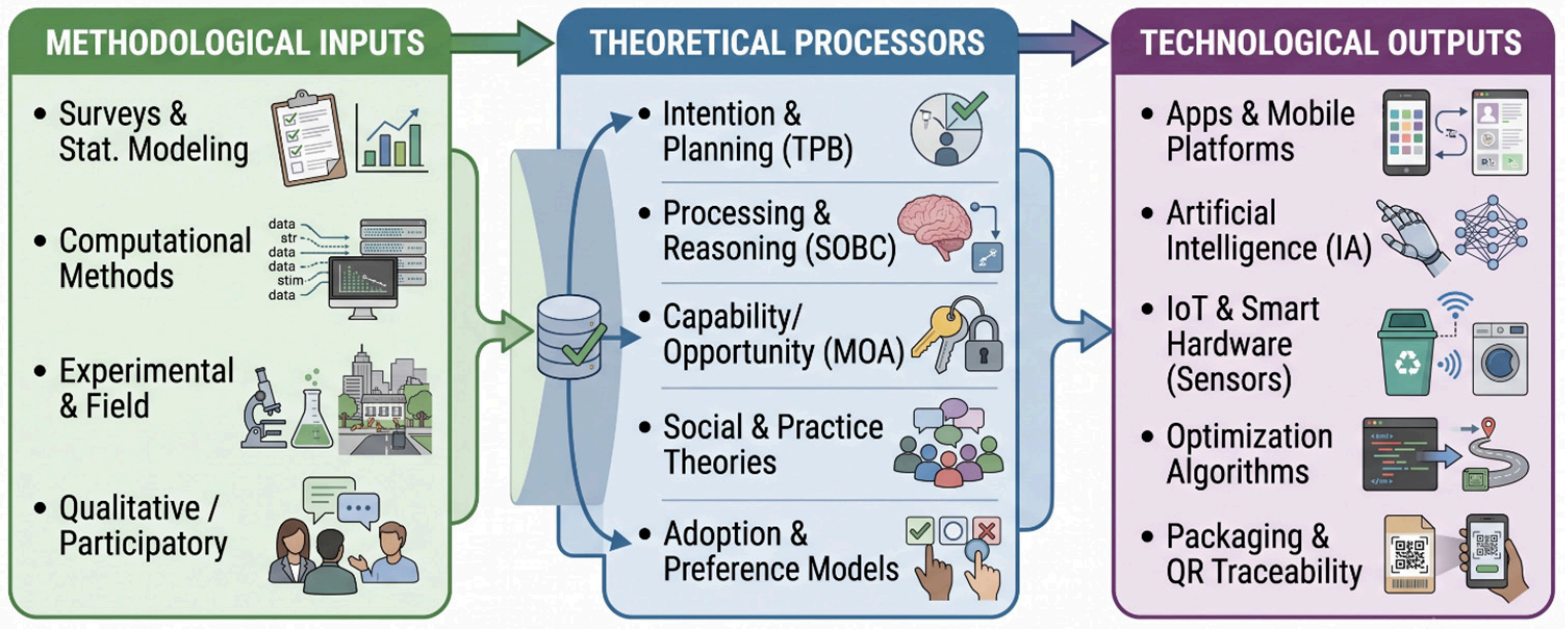
The socio-technical landscape of consumer food waste technologies. Source: Elaborated by the authors with the support of Gemini Notebook for visual formatting purposes only. Conceptual interpretation, theoretical integration, and final validation of the figure were undertaken by the authors.

Figure 4 provides an integrated socio-technical framework as a comprehensive visual synthesis of the methodological and theoretical landscape mapped in Table 2. This diagram untangles the complex interactions between research designs, behavioral models, and technological interventions identified across the 22 reviewed papers. As illustrated in Figure 4, academic literature operates within a dynamic tripartite structure. First, the Methodological Inputs define how consumer FW is captured, contrasting traditional subjective inputs (such as surveys and diaries) with emerging objective tracking methods (such as computational vision, sensor-based weighing, and ethnography). Second, these inputs are filtered through Theoretical Processors. Here, classical linear models (such as the TPB) are expanded by systemic and contextual lenses (such as Social Practice Theory and the MOA framework) to capture non-intentional household routines.

Among the included studies, Gul et al. [44] explicitly invoke Social Practice Theory (SPT) to frame their analysis of AI-driven, participatory approaches to sustainable consumption. Drawing on the original formulation by Shove et al. [87], SPT conceptualizes everyday consumption routines, including food-related practices, as emerging from the interaction of three elements: material infrastructures, embodied competencies, and shared meanings, rather than from individual, purely cognitive deliberation. Gul et al. [44] mobilize this lens to argue that interventions aimed at sustainable consumption should target not only consumers’ attitudes, but also the material and skill-based conditions that make wasteful or non-wasteful practices more or less likely to occur. In this review, we draw on this framing, which is explicitly used within our included sample by [44], as an external interpretive lens to contextualize the conceptual extension of TPB and MOA proposed later in this section.

Finally, this cognitive-behavioral dynamic materializes in specific Technological Outputs. These range from passive mobile platforms and algorithmic tracking to smart packaging and optimization models, all of which actively reshape choice architecture and redirect consumer practices in real time. By visualizing these intersections, Figure 4 moves beyond a descriptive listing of papers to offer a structural map of how technological materiality co-constitutes contemporary food waste behaviors.

Appendix A summarizes the methods, behavioral theories, and technologies applied in consumer FW research.

Table 2 synthesizes the studies that implemented technological innovations without relying on self-report measures and summarizes their principal findings.

A cross-sectional review of these studies indicates that digital tools do more than automate data collection; they fundamentally reshape understanding of FW phenomena. Objective measurement technologies, such as artificial intelligence, sensors, physical audits, and biometric devices, consistently reveal a gap between declared intentions and observed behavior. While surveys typically capture attitudes and social norms, digital observation tools expose the materiality of waste and automatic habits that underlie disposal practices.

Consequently, the evidence suggests that the most promising methodological path is hybridization. Combining user inputs collected via apps with objective validation through AI, sensors, or physical audits offers a robust approach for designing evidence-based interventions. Such mixed methods mitigate self-report bias and provide the precise metrics necessary to evaluate the real impact of sustainability strategies.

### 3.3. Gaps and Implementation Barriers

The extant literature at the intersection of consumer FW and technology, despite its growth, exhibits methodological limitations that constrain the generalizability of its findings. A predominant shortcoming is the heavy reliance on self-reported survey data, which introduces social-desirability bias and recall errors. Studies such as Kör et al. [19] and Jörissen et al. [77] acknowledge that consumer perceptions alone may not reflect actual waste behavior. Similarly, Aloysius et al. [82] and Wiśniewska and Czernyszewicz [83] note discrepancies between stated intentions and observed actions in questionnaire-based analyses, underscoring the need for more objective measurement approaches.

The cross-sectional design of many studies further limits causal inference regarding technological and behavioral relationships. Research on food-delivery apps [35,40,80] often relies on single-time-point data, restricting insight into how sustained technology use shapes long-term habits, such as over-ordering or routines for reusing leftovers.

Sample limitations are also evident: numerous studies focus on homogeneous or non-representative groups, impeding broader extrapolation. For example, Masson et al. [81] conducted kitchen observations with only 20 participants in an artificial setting, potentially altering natural behavior. Gul et al. [44] and Jones-Garcia et al. [18] worked with small samples of students or specific households, and Roe et al. [84] reported a predominantly female, local sample, all of which constrain external validity.

Technological validation and usability present additional barriers. Interventions may fail due to design flaws or user fatigue. Lim et al. [86] identified manual data entry as a major obstacle to long-term app adoption. Hardware issues were reported by Jones-Garcia et al. [18], where condensation in smart-bin cameras compromised data collection, indicating that IoT-based research tools still lack the robustness required for prolonged domestic deployment.

In simulation and modeling domains, advanced algorithms yield powerful insights but often lack large-scale empirical validation. Woolley et al. [73] proposed an optimization algorithm for household inventory management that assumes standardized expiry dates and has not been field-tested in complex real-world scenarios. Ou et al. [74] based dynamic promotion models on historical data, necessitating field verification to confirm whether theoretical waste reductions translate into commercial and household practice.

AI accuracy also requires further attention before broad application. Yu et al. [43] reported nearly 50% discrepancies between consumer-estimated waste weights and AI-calculated weights, while acknowledging that AI models need training on larger, more diverse datasets to ensure reliability. Gul et al. [44] emphasize that pre-trained models may fail to recognize culturally specific foods in emerging-economy contexts, highlighting the need for localized training data and validation.

Digital technologies and AI offer substantial benefits for FW reduction by enhancing inventory control, personalizing menu planning, and enabling automated, real-time waste tracking that allows for swift corrective actions [21]. Empirical evidence highlights that AI functions as a strategic tool for more efficient food management, delivering both economic and environmental benefits [21], and future research should focus on combining these waste-tracking devices with consumer-level interventions to further enhance FW reduction [20]. Crucially, digital and AI-based measures provide greater accuracy than traditional tracking methods. Traditional questionnaires and self-report methods, such as written diaries, have been shown to underestimate household FW and suffer from compromised accuracy [88,89]. In fact, approximately 50% of the error in self-reported intake methods stems from a participant’s inability to accurately estimate served and uneaten portion sizes [89]. In contrast, digital applications like the FoodImage app are perceived more favorably by users, as they are preferred in direct comparisons, present a lower time burden, and are considered to provide superior accuracy [89].

Despite these clear advantages in improving precision, reducing labor, and enabling real-time monitoring, AI systems still face notable drawbacks and limitations. Challenges persist regarding the technology’s ability to adapt to diverse food types, ensure algorithmic fairness, and address data privacy concerns [90]. Furthermore, automated systems remain susceptible to AI hallucinations; therefore, maintaining user agency by allowing individuals to manually modify quantities or rectify misclassifications before data is committed remains a critical requirement for ensuring the ultimate accuracy of quantitative inputs [91].

The proposed methodological and technological transition faces several barriers. Usability and user fatigue remain primary obstacles: although automated data collection is ideal, current technologies often require substantial user participation. Lim et al. [86] and Jones-Garcia et al. [18] show that manual data entry or hardware maintenance disrupts domestic routines and leads to abandonment after the novelty period. Technical failures, such as condensation on smart-bin cameras or sensor drift, compromise long-term data reliability and make sustained domestic deployment financially and logistically challenging [18,84].

A second critical barrier concerns ethical tensions and privacy associated with domestic monitoring. Proposals to use technology probes and AI to analyze fridge and bin contents encounter consumer resistance to intrusive surveillance. Jones-Garcia et al. [18] warn that increased visibility may provoke guilt or shame rather than empowerment, potentially alienating participants. Digital exclusion and inadequate infrastructure, highlighted by Ita et al. [78] and Kör et al. [19], create selection biases, whereby solutions work primarily for affluent, tech-literate populations and fail to represent vulnerable groups or contexts with unstable electricity and internet.

From a commercial perspective, structural conflicts of interest can hinder collaboration among researchers, app developers, and retailers. Sharma et al. [80] and Islam et al. [40] note that many food-delivery and retail business models depend on sales volume; measures that reduce over-ordering or share sales data to curb waste may be perceived as financially disadvantageous, generating corporate resistance to waste-reduction technologies that threaten short-term margins.

Finally, psychological and cultural barriers, such as neophobia toward food technologies, impede adoption. Tadesse et al. [41] found that consumer distrust of novel food technologies can limit uptake of promising solutions like active packaging and smart QR codes. AI-based image recognition faces cultural specificity challenges: Gul et al. [44] highlight that models trained on Western datasets often fail to identify local dishes in emerging economies, necessitating costly retraining to ensure global scalability and cultural relevance. Table 3 summarizes the principal gaps and implementation barriers, with corresponding sources.

## 4. Discussions and Implications

This study shows that the nexus of consumer behavior, technology use and FW should be regarded as an integrated research domain rather than an incidental intersection. By mapping prevailing behavioral theories, methods, and technological innovations applied to measurement and intervention, the review demonstrates how technology simultaneously operates as an explanatory variable, a methodological instrument, and an agent that transforms consumption practices. For consumer-behavior research, this implies greater incorporation of technological materiality into analyses; for technology studies, it reinforces the need for contextualized empirical validation; and for the food waste agenda, it provides a more robust, data-driven methodological orientation grounded in objective measures and evidence-based interventions.

The temporal mapping revealed a shift from predominantly theoretical approaches, centered on cognitive models such as TPB, to studies that integrate multiple data sources and emerging technologies. This transition signals a shift in methodological orientation toward more diverse data sources and technology-enabled measurement, aligned with broader movements in behavioral science and sustainability studies. This shift should not, however, be read as evidence of overall improved methodological rigor since persistent limitations in sample size, representativeness, and study duration constrain the strength of causal and generalizable conclusions. The methods portfolio has diversified substantially, with the rise in AI, machine learning, IoT sensors, computer vision, and technology probes. These tools reduce self-report bias and enable capture of real consumption and disposal behaviors, supporting more accurate diagnostics and the development of just-in-time interventions capable of influencing everyday habits in situated and responsive ways.

The review identified geographic clusters leading research at the technology–food waste interface. The concentration of research output in countries such as Australia, China, Italy, the Netherlands, and the United Kingdom is consistent with these countries’ broader research capacity in sustainability-related fields. However, our bibliometric analysis, based solely on authors’ institutional affiliations, cannot by itself establish alignment among public policy, research investment, and technological innovation. We therefore interpret this concentration as an indicator of research capacity rather than direct evidence of coordinated policy–industry action and call for dedicated policy-focused research to examine this question.

Despite notable advances, important methodological weaknesses persist. The predominance of studies with limited sample diversity, short experimental durations and low demographic representativeness constrains generalizability. Many evaluated technologies remain at the prototype stage, with few studies assessing continued use, long-term acceptability, or sustained intervention efficacy. Implementation barriers identified include usability challenges, poor integration with domestic routines, digital-access inequalities, cultural resistance, ethical concerns about data use and commercial conflicts involving digital platforms. These factors highlight the need for interdisciplinary approaches that address not only technical efficiency but also social, cultural and economic viability.

The synthesized evidence can guide the design of more effective and inclusive technologies by indicating which functionalities are most likely to promote behavioral change, contextual notifications, meaningful impact visualizations, integration with purchasing and food-preparation routines, and personalized, data-driven interventions. These insights are relevant for designers, developers, policymakers, and food-sector organizations seeking to translate technological potential into practical reductions in household FW.

A critical methodological distinction emerged from our review regarding what constitutes objective measurement. It is scientifically inaccurate to treat algorithmic estimation (such as AI-driven computer vision and image-based weight predictions) as equivalent to direct physical measurement (such as direct scale weighing). While AI innovations, such as the GPT-4o image parsing utilized by Yu et al. [43], offer a low-cost and scalable alternative to traditional diaries, they remain estimations. Indeed, Yu et al. [43] documented a significant 50% discrepancy between consumer self-assessment and AI weight estimation. However, because AI predictions are susceptible to algorithmic hallucinations, spatial distortions in photos, and food density variations, algorithmic estimation must not be equated with objective physical reality without rigorous, baseline calibration. Direct physical weighing [34,86] remains the absolute gold standard against which any machine learning model must be calibrated.

### 4.1. Technology-Mediated Theoretical Extension: Redefining TPB and MOA Through Human–Computer Interaction (HCI)

Traditional FW literature has predominantly relied on static, individual-centric cognitive models, most notably Ajzen’s [92] TPB and Ölander and Thøgersen’s [93] MOA framework [36,77,82]. Within these traditional paradigms, technology is typically treated as an external, passive variable, a peripheral channel for information delivery, or a static infrastructural facilitator [77,78]. However, as consumer food practices are increasingly mediated by digital interfaces, platforms, and connected appliances, this passive view becomes insufficient. This study contributes to the literature by integrating the perspectives of HCI and social practice theories [18,86].

Within the classic TPB, subjective norms represent the perceived social pressure from an individual’s immediate, physically proximate circle (e.g., family, friends, or cohabitants) to perform or abstain from a specific behavior [35,92]. In the digital era, however, the proliferation of food-sharing platforms, mobile applications, and online communities significantly scales and intensifies these normative pressures [83,86,94]. Rather than relying on abstract, unquantified social expectations, digital anti-waste platforms introduce structural mechanisms such as gamification, public feedback, and digital social rankings [34,86].

These digital architectures shift subjective norms from private, reflective cognitions to a dynamic landscape of social surveillance and mutual reflection [86]. For example, technologies like smart bins or mobile diaries that automatically upload images and waste metrics to shared networks (e.g., Facebook or specialized community apps) expose private domestic waste behaviors to a broader collective [18,86]. This digital exposure activates deep-seated desires for social acceptance and triggers continuous comparison processes [86]. Subjective norms are thus influenced by digital behavior, in which the individual’s moral norms and self-regulation are co-steered and audited by algorithmic feedback and reputational rankings [80,86]. The subjective norm is no longer a static antecedent of intention; it is a highly visible, community-regulated digital feedback loop that actively co-regulates household practices [83,86].

Under the MOA framework, opportunity is classically conceptualized as a set of external, static, and situational circumstances, such as time constraints, infrastructure limitations, or daily schedule disruptions, that either facilitate or hinder the execution of a practice [82,93]. The integration of smart kitchen systems, IoT, and connected sensors fundamentally transforms this passive construct of opportunity into a dynamic, real-time intervention space [18,73].

By automating domestic inventory tracking and employing Just-in-Time Adaptive Interventions, technology-aided, tailored sustainability interventions actively restructure the user’s choice architecture [84]. When smart refrigerators or automated inventory apps track food decay and send real-time push notifications suggesting tailored recipes for ingredients nearing expiration, the traditional barrier of “lack of opportunity” is neutralized [73,84].

This shift reframes the construct of opportunity as cognitive accessibility or behavioral availability [84,95]. Instead of requiring high cognitive effort and deliberate planning from the consumer to track and manage leftovers, the smart home ecosystem assumes a portion of the user’s cognitive load [18,73]. By delivering actionable, hyper-personalized solutions precisely when the decision to discard or consume is made, IoT technologies convert a static situational constraint into an algorithmically facilitated action space [73,84]. Consequently, opportunity is no longer merely an external condition the consumer must navigate; it is a fluid, co-constituted state of human–machine collaboration that optimizes resources with minimal cognitive friction [73,86].

By viewing these dynamics through an HCI lens, researchers can move past the “application level” of technology to a level of theoretical evolution [18,86]. This extension bridges cognitive psychology with practice-oriented frameworks, recognizing that intentions and habits are not purely internal mental states but are actively co-shaped by the material affordances of the technologies we use [86,96]. Understanding FW behavior in the digital age requires models that treat technological materiality as a relevant actor influencing domestic practices [18,35,86].

### 4.2. Future Research Agenda

To advance knowledge, the research agenda should prioritize mixed-methods and objective measurement approaches that overcome reliance on self-reports. Integrating physical waste audits with digital interventions is essential to validate the real effectiveness of technologies, as suggested by Soma et al. [34] and Roe et al. [84]. Combining technology probes and digital ethnography with quantitative analyses can yield a more holistic understanding of waste practices, capturing not only what is discarded but also how and why disposal occurs.

Studies must also broaden their geographic and cultural scope to examine how technologies interact with diverse social norms and infrastructures. Wicaksono et al. [42] and Tadesse et al. [41] emphasize the need to test circular-economy models and innovation acceptance across national contexts to capture heterogeneity in consumer preferences. Comparative research between developed and developing countries, as proposed by Kör et al. [19], is crucial for designing technological solutions that are globally relevant yet locally adaptable.

Longitudinal designs are essential for assessing the sustainability of technology-induced behavior changes. Field experiments of extended duration are needed to determine whether interventions such as gamification or tracking apps retain effectiveness after novelty effects dissipate [34,76]. Such designs will clarify habit formation and the persistence of sustainable routines.

Automating data collection should be a central development goal to reduce user burden and improve data quality. Lim et al. [86] and Woolley et al. [73] advocate for systems that automatically integrate purchase data (digital receipts) and household inventory, using AI to predict waste and recommend actions without constant manual input. This would shift technology from a passive recording tool to an active domestic management assistant.

A promising frontier is the study of emotional and psychological responses to waste-prevention technologies. Research should investigate how visual feedback and monitoring (for example, camera-equipped bins) influence guilt, moral norms and privacy perceptions. Jones-Garcia et al. [18] and Islam et al. [40] argue that understanding the interplay between the technological visibility of waste and consumers’ emotional reactions is vital to designing interventions that engage rather than alienate users.

Research should also explore the integration of technology across supply chains and retail. Ou et al. [74] and Tadesse et al. [41] propose examining how retail innovations, dynamic pricing, and smart packaging with QR codes affect subsequent household behavior. Future studies ought to trace information flows from retailer to consumer to test whether enhanced traceability effectively reduces post-purchase waste. Deepening analysis of unstructured data with big data and machine learning will capture behavioral nuances at scale. Shinde et al. [79] and Ita et al. [78] recommend data-mining techniques to identify consumption patterns and rebound effects that surveys miss. Refining algorithms to process images and social-media text can provide near-real-time insights into emerging attitudes and sustainable consumption practices.

It is also essential to investigate the role of digital infrastructure and technological literacy as prerequisites for solution efficacy. Ita et al. [78] and Wiśniewska and Czernyszewicz [83] note that home smartification depends on digital skills and infrastructure. Research should assess how digital exclusion limits reach and propose inclusive designs that serve diverse demographics, including older adults and low-income populations.

Advancing the field requires shifting from descriptive, intention-based studies to experimental, longitudinal, and data-driven research. Integrating smart hardware, advanced analytics and robust behavioral theories will enable precise measurement and effective intervention in household routines. This integrated approach is essential to translate the potential of digital technologies into tangible reductions in food waste and to align consumer behavior with global sustainability objectives. Table 4 summarizes the main research recommendations with corresponding sources.

The MMAT-based appraisal provides a structured complement to the thematic limitations discussed throughout this review. Consistent with MMAT developer recommendations, ratings were not aggregated into a composite score nor used to exclude studies; rather, they offer a transparent, criterion-level account of the methodological characteristics underlying the evidence synthesized in this SLR. The recurring uncertainty regarding sample representativeness and non-response bias across the quantitative descriptive studies, which constitute the largest methodological cluster in this review, reinforces the recommendation for demographically diverse, representative sampling strategies (Table 3). Similarly, the limited empirical validation observed among computational and simulation-based studies substantiates the call for field-testing algorithmic models under real-world conditions before their integration into consumer-facing interventions. We note that this appraisal was conducted after the final sample of 22 studies was established, following completion of the inclusion/exclusion screening process; its purpose was methodological transparency and characterization, not sample refinement.

### 4.3. Implications and Limitations

The study advances theory by reframing the relationship between technology and consumer FW. We found that technology operates simultaneously as an explanatory variable, a methodological instrument, and an active agent of behavioral change. By systematically mapping prevailing frameworks alongside practice-theoretical and social-practice perspectives, the review clarifies how cognitive, situational, and material determinants interact in technologically mediated contexts related to FW. This synthesis presents guidance for future work that seeks to link micro-level psychological constructs with meso-level technological affordances and macro-level infrastructures.

To practitioners, this review provides a clear, evidence-based roadmap for researchers, designers, and policymakers by identifying robust methodological priorities and implementation constraints. It recommends hybrid, objective measurement strategies, longitudinal field trials to assess habit formation, and automated data pipelines to reduce user burden. The study also pinpoints critical barriers (usability, user fatigue, privacy and ethical concerns, and psychological and cultural barriers) and highlights the geographic concentration of scientific output in a small number of countries, which may offer transferable lessons for contexts with weaker research infrastructure, although the bibliometric data alone do not permit direct inference about underlying policy or industry alignment. Collectively, these contributions translate into actionable guidance for designing inclusive, empirically validated technologies and policy instruments capable of producing measurable reductions in consumer FW.

Reducing FW requires coordinated action across the food system rather than isolated interventions targeting individual consumers or organizations. Effective collaboration among policymakers, researchers, retailers, suppliers, hospitality businesses, and consumers can improve communication and information sharing, promote shared responsibility, align incentives, and support evidence-based prevention measures throughout the supply chain [28,97,98,99]. In this context, trade associations can contribute by fostering a common sustainability vision and providing platforms for reducing the industry’s environmental impacts. Stronger collaboration across disciplinary perspectives is also essential, given the complexity of FW [28,97].

FW should also be addressed as early as possible in the supply chain, since waste at the consumption stage can be influenced by practices adopted by upstream actors. Accordingly, policies should be integrated with broader measures concerning food, agriculture, food standards, food poverty alleviation, and sustainable production and consumption, rather than individualizing responsibility. A coordinated strategy involving actors from production to consumption may therefore help translate research findings into scalable policies, business practices, and digital solutions for FW prevention [28,99].

To synthesize these findings into an actionable framework, Table 5 maps the consumer FW journey against the technology touchpoints, measurement approaches, and intervention mechanisms identified across the reviewed literature. The table traces five sequential stages—planning/purchasing, storage, preparation, consumption, and disposal—and identifies, for each stage, the dominant technology type (shopping-list apps, smart fridges/IoT sensors, recipe-suggestion apps, portion-tracking apps, and computer-vision-enabled smart bins), the measurement approach employed (self-report, sensor-based capture, or algorithmic estimation versus direct physical weighing), and the corresponding behavioral mechanism activated (norm-based social comparison, JITAI-driven opportunity facilitation, or personalized/gamified nudges).

This mapping makes explicit two patterns that remain implicit in narrative synthesis alone. First, opportunity-based mechanisms, consistent with the MOA reconceptualization previously discussed, concentrate specifically in the storage and preparation stages, where IoT sensors and inventory-aware recommendation systems reduce cognitive load through real-time, situational facilitation. Norm-based and social-comparison mechanisms, by contrast, bookend the journey, appearing at the planning stage, where shopping decisions are shaped by social expectations, and again at the disposal stage, where gamified feedback and public waste metrics reactivate subjective-norm dynamics. Second, measurement rigor is unevenly distributed: self-reported data continue to dominate the planning, preparation, and consumption stages, while more objective approaches, sensor-based tracking and algorithmic estimation, are concentrated almost exclusively at the disposal stage, precisely where the discrepancy between algorithmic estimation and direct physical weighing discussed earlier becomes most consequential for research validity. Notably, the technologies populating the storage and disposal stages are also those most frequently associated with the geographic clusters identified earlier, raising the possibility, though not directly evidenced by this bibliometric mapping, that research capacity in these countries has disproportionately advanced sensor- and vision-based innovation relative to normatively oriented, planning-stage interventions. This uneven distribution also intersects with the incentive-alignment concerns raised throughout this review: because retailers and app-based platforms have direct commercial visibility and control over the planning and purchasing stage, the relative scarcity of objective measurement tools at this stage may reflect not only a technical gap but also a misalignment between platform business incentives and food-waste-reduction goals. Taken together, these patterns reinforce the need for the methodological and empirical advances discussed throughout this review, while also underscoring the boundaries within which the present synthesis should be interpreted.

This SLR is not free of limitations. While a sample size of 22 papers might be considered relatively compact, the literature syntheses in emerging or interdisciplinary domains inherently face a limited pool of primary empirical evidence [100]. Preserving a narrow, high-standard screening protocol is essential to protect the internal validity of the review; expanding the criteria merely to increase the absolute volume introduces the risk of including lower-quality, tangential data that diminishes the reliability of the conclusions [70]. Therefore, this tight selection serves as an objective structural finding that maps a distinct research gap and field [49].

Additionally, nearly half of the included studies were published across only three journals (*Sustainability, British Food Journal*, and *Food Quality and Preference*), with Sustainability alone accounting for four of the 22 studies (18%). This concentration may reflect editorial scope alignment rather than a genuine field-wide consensus and raises the possibility of outlet-specific selection or framing effects (e.g., a preference for sustainability-framed contributions). Readers should interpret thematic patterns with this potential concentration bias in mind.

Although rigorous methods were applied, the predefined search scope, restricted to selected databases and search terms, may have omitted relevant studies published in emerging journals, gray literature, or non-indexed articles. Heterogeneity in study designs, metrics, technologies and sociocultural contexts complicated direct comparisons and precluded quantitative synthesis. The predominance of research from high-income countries limits the applicability of findings to contexts with greater socioeconomic inequality or limited digital infrastructure.

## 5. Conclusions

This systematic review of 22 peer-reviewed studies confirms that digital technology has moved from a peripheral variable to a structural component in consumer FW research. The literature reveals a clear methodological split: quantitative studies relying on cross-sectional surveys and statistical modeling, where technology is treated as an explanatory factor, versus experimental and design-oriented studies that deploy technology as a direct behavioral intervention (e.g., apps, smart sensors, feedback tools). Research remains geographically concentrated in Australia, China, Italy, the Netherlands, and the United Kingdom, and is published predominantly in journals focused on environmental sustainability, food chain management, and consumer psychology, indicating both thematic maturity and a need for broader contextual diversification.

Despite this growth, the evidence base remains methodologically constrained. Most studies depend on self-reported measures, which are vulnerable to recall bias and social desirability effects, and predominantly adopt cross-sectional designs that preclude causal inference or assessment of long-term behavior change. These limitations restrict confidence in the real-world effectiveness of the technologies reviewed and point to a validation gap between technological promise and empirical proof of impact.

Future research should prioritize three specific methodological shifts: (1) longitudinal designs capable of tracking habit formation and persistence beyond initial intervention exposure; (2) objective behavioral measurement to complement or replace self-report instruments; and (3) mixed-methods approaches combining quantitative outcome measures with qualitative exploration of users’ emotional and psychological responses to FW-prevention technologies, including fatigue, trust, and perceived surveillance. Additionally, research should examine adoption barriers tied to usability, privacy concerns, and cultural context, particularly through cross-national comparative studies that test whether the effectiveness of interventions generalizes beyond the current geographic concentration of evidence.

In addition, future research and industry practice should examine incentive-alignment mechanisms that reconcile the commercial objectives of food retailers and app-based platforms with food-waste-reduction goals. Because these actors may be incentivized to maximize sales and purchasing frequency, interventions should explore how business performance indicators, revenue models, and platform recommendations might also reward accurate demand forecasting, appropriate purchasing, surplus redistribution, and the sale of products before they become waste. Such mechanisms could include shared performance targets, financial or reputational incentives, and digital tools that support more accurate consumer decisions while maintaining commercial viability.

## Figures and Tables

**Table 1:** Scientific production—journals.

Journal	Number of Papers Published	References
*Sustainability*	4	Jörissen et al. [77] Soma et al. [34]. Ou et al. [74]. Gul et al. [44].
*British Food Journal*	3	Kör et al. [19] Wiśniewska and Czernyszewicz [78]. Islam et al. [40].
*Food Quality and Preference*	3	Masson et al. [81] Jaeger et al. [85] Aloysius et al. [77].
*Cleaner Environmental Systems*	1	Wicaksono et al. [42]
*Foods*	1	Jones-Garcia et al. [18]
*International Journal of Hospitality Management*	1	Sharma et al. [80]
*International Journal of Human-Computer Studies*	1	Lim et al. [86]
*Journal of Cleaner Production*	1	Tadesse et al. [41]
*Journal of Environmental Management*	1	Shinde et al. [79]
*Quality & Quantity*	1	Yu et al. [43]
*Resources, Conservation & Recycling*	1	Roe et al. [84]
*Sensors*	1	Valpreda and Zonda [76]
*Sustainable Production and Consumption*	1	Woolley et al. [73]
*Technovation*	1	Shankar et al. [35]
*Urban Climate*	1	Ita et al. [78]

Source: Elaborated by the authors based on the literature reviewed.

**Table 2:** Technological innovations and measurement methods (non-self-reported).

Reference	Technological Innovation	Measurement Type	Measurement Method (Non-Self-Reported)	Main Findings
Gul et al. [44]	Participatory visual methods (Photovoice) coupled with an automated AI pipeline (convolutional neural network—MobileNetV2).	Algorithmic Estimation	Automated computer vision object and theme classification of participant-generated food waste photographs.	The combination of Photovoice with MobileNetV2 image processing provided scalable, non-intrusive insights into daily consumption routines, demonstrating that visual indicators trigger high cognitive reflection.
Yu et al. [43]	REGUSTO mobile application integrated with generative AI (GPT-4o vision model via LangChain framework).	Algorithmic Estimation	Computer vision weight estimation and automated ingredient/portion description from user-uploaded leftovers photos.	Revealed a significant (~50%) discrepancy between consumer self-assessment and AI-estimated portion weights, highlighting that AI estimations must be calibrated against physical weighing baselines.
Soma et al. [34]	Information-based campaign augmented with an online quiz game featuring points, trivia, and rewards.	Direct Physical Measurement	Curbside waste audits (collection, manual sorting, and physical weighing of household organic garbage bags).	Gamification and passive informational interventions significantly increased self-reported waste awareness, but did not translate into a statistically significant reduction in audited household FW.
Jones-García et al. [18]	Smart household bin probe equipped with an integrated load-cell scale, a Raspberry Pi, and a digital camera.	Hybrid/Direct Physical Measurement	Automatic physical weighing synchronized with context photography triggered at each bin lid-opening event.	The IoT probe successfully materialized “invisible” routine discarding behavior, triggering strong affective self-reflection (e.g., guilt and shame) upon visual confrontation.
Lim et al. [86]	Community-based “Social Recipes” mobile app integrated with an augmented household smart bin.	Direct Physical Measurement	Automatic logging of discarded food weight in situ using a digital scale connected to a Raspberry Pi Wi-Fi module.	Social comparison feedback (eco-feedback displaying real-time waste weights relative to peer households) was highly effective in reducing waste, outperforming the informational recipe-sharing feature.
Jaeger et al. [85]	High-resolution digital imaging of fruit defects paired with wearable, non-invasive eye-tracking biometric hardware.	Biometric/Behavioral Measurement	Continuous tracking of consumers’ eye movements (time to first fixation, fixation count, visit count) when evaluating apples with defects.	Defect severity directly increased visual attention and cognitive load (fixation count), triggering rapid, low-effort rejection heuristics at the point of purchase and consumption.
Masson et al. [81]	Controlled, standardized mock kitchen laboratory equipped with five ceiling-mounted and portable GoPro digital cameras.	Direct Observational Measurement	Video recording and frame-by-frame behavioral coding of spatial and temporal product movement during food storage tasks.	Uncovered three distinct grocery-sorting strategies among consumers and quantified significant behavioral deviations from official food safety and refrigerator storage guidelines.
Roe et al. [84]	FoodImage smartphone app utilized within a Technology-Aided Tailored Sustainability Intervention (TATSI).	Algorithmic-Manual Estimation	Digital photography of food selection, consumption, and leftovers, with portion weights coded manually by trained researchers.	The tailored mobile intervention yielded a statistically significant 78.8% reduction in plate waste, showing the efficacy of passive image capture and customized feedback.

Source: Elaborated by the authors based on the literature reviewed.

**Table 3:** Summary of gaps and implementation barriers in consumer FW and technology research.

Gap	Implementation Barriers	References
Self-Report Bias: Overreliance on subjective, memory-dependent diaries or surveys that systematically underreport actual household FW.	High financial costs of hardware (IoT devices/sensors) and operational failures in natural home settings (e.g., camera lens condensation from hot food waste).	Aloysius et al. [82]; Kör et al. [19]; Soma et al. [34]; Yu et al. [43]
Lack of Causality: Prevalence of cross-sectional (cross-sectional) designs that capture only temporary correlations rather than long-term behavioral changes.	High user attrition/dropout rates and fatigue during extended research protocols; difficulty in isolating external confounding factors in uncontrolled kitchens.	Islam et al. [40]; Shankar et al. [35]; Sharma et al. [80]
Limited and Homogeneous Samples: Overrepresentation of highly educated, narrow sociodemographic cohorts (e.g., students/women) and artificial test kitchens.	Digital divide/exclusion among vulnerable, low-income, or elderly populations who lack access to necessary hardware, plus high logistics and recruitment costs.	Gul et al. [44]; Masson et al. [81]; Roe et al. [84]; Wicaksono et al. [42]
Usability Friction and Data Fatigue: High cognitive load and user effort of manually inputting household pantry inventory and daily discard logs in apps.	Severe technical challenges regarding database interoperability between commercial retailers and independent, third-party household management apps.	Lim et al. [86]; Woolley et al. [73]; Jones-Garcia et al. [18]
Algorithmic Equivalence Bias (AI vs. Ground Truth): Treating AI-driven visual weight estimates as absolute measurements rather than statistical approximations.	Algorithmic cultural bias (difficulty in identifying mixed or regional dishes) and the user burden of placing standardized spatial reference cards in every daily photo.	Yu et al. [43]; Gul et al. [44]; Ou et al. [74]
Geographic and Infrastructural Constraints: Extreme scarcity of comparative cross-cultural studies, with research heavily concentrated in developed Western economies.	Basic infrastructure instability (e.g., frequent power outages, slow or expensive mobile internet) that makes continuous IoT monitoring unfeasible in emerging regions.	Kör et al. [19]; Tadesse et al. [41]; Wicaksono et al. [42]
Temporary Novelty Effect: Short intervention windows (<4 weeks) that fail to determine if waste-reduction practices survive once user enthusiasm fades.	Sharp drop-off in user engagement after the first month; difficulty in sustaining intrinsic motivation without continuous financial, social, or gamified rewards.	Soma et al. [34]; Valpreda and Zonda [76]
Supply Chain Actor Fragmentation: Focusing exclusively on consumer behavior in isolation, ignoring supplier or retail-side technological links.	Structural commercial conflicts of interest, as retail and online food delivery business models often rely on inducing consumer over-ordering (over-ordering).	Ou et al. [74]; Tadesse et al. [41]; Woolley et al. [73]
Under-Explored Psychological and Privacy Factors: Neglecting the emotional impact of tech feedback and user anxiety regarding smart surveillance.	Consumer resistance to intrusive surveillance in intimate and morally charged spaces (refrigerators, trash cans) and neophobia toward novel packaging tech.	Islam et al. [40]; Jaeger et al. [85]; Sharma et al. [80]

Source: Elaborated by the authors based on the literature reviewed.

**Table 4:** Summary of future research recommendations in consumer FW and technology research.

Suggestion for Future Research	References
Deploy direct physical measurement methods (such as in situ load cell scales or garbage-bag waste audits) combined with visual diaries for robust data triangulation.	Aloysius et al. [82]; Kör et al. [19]; Soma et al. [34]; Yu et al. [43]
Conduct longitudinal field experiments and randomized controlled trials (RCTs) to track habit formation and intervention decay over time.	Islam et al. [40]; Shankar et al. [35]; Sharma et al. [80]
Broaden research boundaries to demographically diverse, representative samples and examine behaviors in natural household environments.	Gul et al. [44]; Masson et al. [81]; Roe et al. [84]; Wicaksono et al. [42]
Transition toward passive, automated data collection (e.g., direct integration of retail e-receipts with inventory systems or passive image scanning).	Lim et al. [86]; Woolley et al. [73]; Jones-Garcia et al. [18]
Formally separate direct physical measurement from algorithmic estimation. Mandate in-laboratory physical calibration as a baseline prerequisite to correct AI model errors before deployment.	Yu et al. [43]; Gul et al. [44]; Ou et al. [74]
Undertake cross-cultural studies focusing on localized food practices and infrastructural vulnerabilities in the Global South.	Kör et al. [19]; Tadesse et al. [41]; Wicaksono et al. [42]
Conduct extended-timeline studies (>12 to 24 months), incorporating systematic post-intervention assessments (removing the technology completely).	Soma et al. [34]; Valpreda and Zonda [76]
Investigate vertical technological integration, linking retail tech (dynamic pricing, smart packaging, QR traceability) directly to household storage practices.	Ou et al. [74]; Tadesse et al. [41]; Woolley et al. [73]
Explore how visual eco-feedback (e.g., smart camera bins) triggers moral self-regulation (guilt/shame), and address psychological barriers to smart-home monitoring.	Islam et al. [40]; Jaeger et al. [85]; Sharma et al. [80]

Source: Elaborated by the authors based on the literature reviewed.

**Table 5:** Consumer food waste journey: technology touchpoint map.

Stage	Plan/Buy	Store	Prepare	Consume	Discard
Technology type	Shopping list apps (MOA: opportunity)	Smart fridges/IoT sensors (decay alerts)	Recipe suggestion apps (MOA: opportunity)	Portion tracks apps	Smart bins/computer vision (measurement)
Measurement type	Self-report	Sensor-based	Self-report	Self-report	Algorithmic estimation vs. Direct weighing
Intervention mechanism	Norm-based (social comparison)	JITAI push notifications	Personalized nudges	Gamified social comparison	Feedback/social comparison

Source: Elaborated by the authors based on the reviewed literature. Note: This journey mapping primarily reflects household consumer contexts; institutional and hospitality settings may follow different sequences and touchpoints not fully captured here.

## Data Availability

No new data were created or analyzed in this study. Data sharing is not applicable to this article. PRISMA 2020 checklist is included in the Supplementary Material.

## Supplementary Materials

The following supporting information can be downloaded at https://www.mdpi.com/article/10.3390/foods15183228/s1: Table S1: PRISMA 2020 checklist.

## Abbreviations

The following abbreviations are used in this manuscript:

AI	Artificial intelligence
FAB	Food sharing Attitudes and Behaviors
FW	Food waste
IoT	Internet of Things
ML	Machine Learning
MOA	Motivation–Opportunity–Ability
PRISMA	Preferred Reporting Items for Systematic Reviews and Meta Analyses
SORC	Stimulus Organism Response Consequence
SLR	Systematic Literature Review
WoS	Web of Science

## Appendix A. Methods, Behavioral Theories and Technologies Applied to Research on Food Waste by Consumers

	**Research Methods**				**Theory**					**Technology**					
**Reference**	**Surveys and Statistical Modeling**	**Computational Methods**	**Experimental and Field Studies**	**Qualitative and Participatory Methods**	**Intention and Planning Models**	**Processing and Reasoning Models**	**Capability and Opportunity Models**	**Social and Practice Theories**	**Adoption and Preference Models**	**Apps and Mobile Platforms**	**Artificial Intelligence (AI) and Machine Learning**	**IoT, Sensors and Smart Hardware**	**Optimization and Management Algorithms**	**Packaging and Traceability Innovations**	**Gamification**
Aloysius et al. [82]	X						X								
Gul et al. [44]				X							X				
Islam et al. [40]	X					X				X					
Ita et al. [78]	X								X					X	
Jaeger et al. [85]				X								X			
Jones-Garcia et al. [18]				X				X				X			
Jörissen et al. [77]	X											X			
Kör et al. [19]	X									X					
Lim et al. [86]				X				X		X					
Masson et al. [81]				X									X		
Ou et al. [74]		X											X		
Roe et al. [84]			X		X					X					
Shankar et al. [35]	X				X					X					
Sharma et al. [80]	X					X				X					
Shinde et al. [79]		X									X				
Soma; Li; Maclaren [34]				X			X								X
Tadesse et al. [41]			X						X					X	
Valpreda; Zonda [76]				X					X			X			
Wicaksono et al. [42]		X									X				
Wiśniews; Czernyszewicz [83]	X								X	X					
Woolley et al. [73]		X											X		
Yu et al. [43]			X							X	X				
Source: Elaborated by the authors based on the literature reviewed.															

## Appendix B. Methodological Quality Appraisal of Included Studies Using MMAT

**Reference**	**MMAT Category**	**S1**	**S2**	**Criterion 1**	**Criterion 2**	**Criterion 3**	**Criterion 4**	**Criterion 5**
Aloysius et al. [82]	4—Quant. descriptive	Y	Y	Y	Y	Y	CT	Y
Gul et al. [44]	5—Mixed methods	Y	Y	Y	Y	Y	CT	Y
Islam et al. [40]	4—Quant. descriptive	Y	Y	Y	N	Y	CT	Y
Ita et al. [78]	4—Quant. Descriptive	Y	Y	Y	N	Y	CT	Y
Jaeger et al. [85]	3—Quant. non-randomized	Y	Y	N	Y	Y	Y	Y
Jones-Garcia et al. [18]	5—Mixed methods	Y	Y	Y	Y	CT	**N**	Y
Jörissen et al. [77]	4—Quant. descriptive	Y	Y	Y	N	Y	CT	Y
Kör et al. [19]	5—Mixed methods	Y	Y	N	N	N	CT	N
Lim et al. [86]	5—Mixed methods	Y	Y	Y	Y	Y	Y	CT
Masson et al. [81]	4—Quant. descriptive	Y	Y	Y	Y	Y	CT	Y
Ou et al. [74]	N/A—Computational	Y	N	—	—	—	—	—
Roe et al. [84]	2—RCT	Y	Y	Y	Y	N	Y	N
Shankar et al. [35]	4—Quant. descriptive	Y	Y	Y	N	Y	CT	Y
Sharma et al. [80]	4—Quant. descriptive	Y	Y	Y	N	Y	CT	Y
Shinde et al. [79]	4—Quant. descriptive	Y	Y	Y	Y	Y	CT	Y
Soma; Li; Maclaren [34]	5—Mixed methods	Y	Y	Y	CT	Y	Y	CT
Tadesse et al. [41]	4—Quant. descriptive	Y	Y	Y	Y	Y	CT	Y
Valpreda and Zonda [76]	1—Qualitative	Y	CT	Y	CT	CT	N	CT
Wicaksono et al. [42]	4—Quant. descriptive	Y	Y	Y	CT	Y	CT	Y
Wiśniewska and Czernyszewicz [83]	4—Quant. descriptive	Y	Y	Y	CT	Y	N	Y
Woolley et al. [73]	N/A—Computational	Y	CT	—	—	—	—	—
Yu et al. [43]	4—Quant. descriptive	Y	Y	Y	N	CT	CT	Y
Source: Elaborated by the authors based on the literature reviewed.								

### Adapted Appraisal of Computational/Algorithmic Studies

C1—Are the model’s underlying assumptions and input parameters clearly and transparently reported?
C2—Is the data used to build/train the model (real, synthetic, or secondary) clearly identified and justified?
C3—Is the model’s output validated against real-world or empirical benchmarks?
C4—Are the limitations of generalizing simulated/modeled results to real consumer behavior explicitly discussed?

**Reference**	**C1 (Assumptions Transparent)**	**C2 (Data Provenance Clear)**	**C3 (Empirically Validated)**	**C4 (Limitations Discussed)**
Ou et al. [74]	Y	Y	N	CT
Woolley et al. [73]	Y	Y	N	Y
